# Rhino-orbital-cerebral mucormycosis caused by *Rhizopus arrhizus* diagnosis via metagenomics next-generation sequencing: a case report

**DOI:** 10.3389/fcimb.2024.1375058

**Published:** 2024-07-16

**Authors:** Jianhong Li, Yuwen Ge, Chengqi Xin, Li Jiang

**Affiliations:** ^1^ Emergency Department, The First Affiliated Hospital of Dalian Medical University, Dalian, Liaoning, China; ^2^ Stem Cell Clinical Research Center, The First Affiliated Hospital of Dalian Medical University, Dalian, Liaoning, China; ^3^ Department of gene detection, Dalian Innovation Institute of Stem Cell and Precision Medicine, Dalian, Liaoning, China

**Keywords:** rhino-orbital-cerebral mucormycosis, metagenomics next-generation sequencing, *Rhizopus arrhizus*, fungal infection, diagnosis

## Abstract

Rhino-orbital-cerebral mucormycosis (ROCM) is a rare, invasive, and fatal fungal disease that is often easily misdiagnosed in the early stages due to the lack of specific clinical manifestations and adequate auxiliary examinations. Early diagnosis and timely therapy are essential for successful treatment. In this report, we presented a 46-year-old man with diabetes who experienced gradual vision loss, right ptosis, swelling, and headaches that progressively worsened to death within 4 days after admission. It was finally confirmed as a fungal *Rhizopus arrhizus* infection by metagenomics next-generation sequencing (mNGS). Our report has proved that mNGS testing should be strongly recommended in highly suspected patients.

## Introduction

Mucormycosis is a lethal, opportunistic infection disease caused by fungi of the order Mucorales, which primarily occurs in immunocompromised individuals with uncontrolled diabetes, ketoacidosis, hematological malignancies, allogeneic stem cell transplantation, and even coronavirus disease 2019 (COVID-19) infection (Bays and Thompson, 2019; Cornely et al., 2019; Chegini et al., 2020; Skiada et al., 2020; Dave et al., 2022). The diagnosis of mucormycosis is difficult and often delayed, while the disease progresses rapidly. Suspected cases need urgent intervention because of the rapidly progressive and destructive nature of the infection with high mortality. Mucormycosis is usually classified into the following clinical forms according to the specific clinical manifestations: pulmonary, cutaneous and soft-tissue, rhino-orbito-cerebral, gastrointestinal, renal, and abdominal (Cornely et al., 2019).

Rhino-orbital-cerebral mucormycosis (ROCM) is a common form of mucormycosis and typically develops in patients with uncontrolled diabetes, which usually originates from the paranasal sinus and subsequently invades the orbit, eye, and brain tissue (Bae et al., 2012; Vallverdu Vidal et al., 2017; Cornely et al., 2019). In the past few years, the incidence rate of this rare disease has been rising with advances in experimental and imaging systems, particularly in India and the Middle East in the COVID-19 era (Skiada et al., 2020; Azhar et al., 2022; Rudrabhatla et al., 2022; Abdorahimi et al., 2023; B et al., 2023).

In this case study, we present a 46-year-old man with diabetes who had a 3-day history of right ptosis, swelling, and headache and worsened to death only 4 days after presentation. Both metagenomics next-generation sequencing (mNGS) and targeted NGS (tNGS) have confirmed the diagnosis of ROCM in blood with *Rhizopus arrhizus* infection.

## Case presentation

A 46-year-old man was hospitalized in the First Affiliated Hospital of Dalian Medical University on 4 October 2023, with a 3-day vision loss in the right eye and swelling, accompanied by a headache. Loss of vision in the right eye was gradual in onset and progressive. His medical history was poorly controlled diabetes and gout.

**Figure 1:**
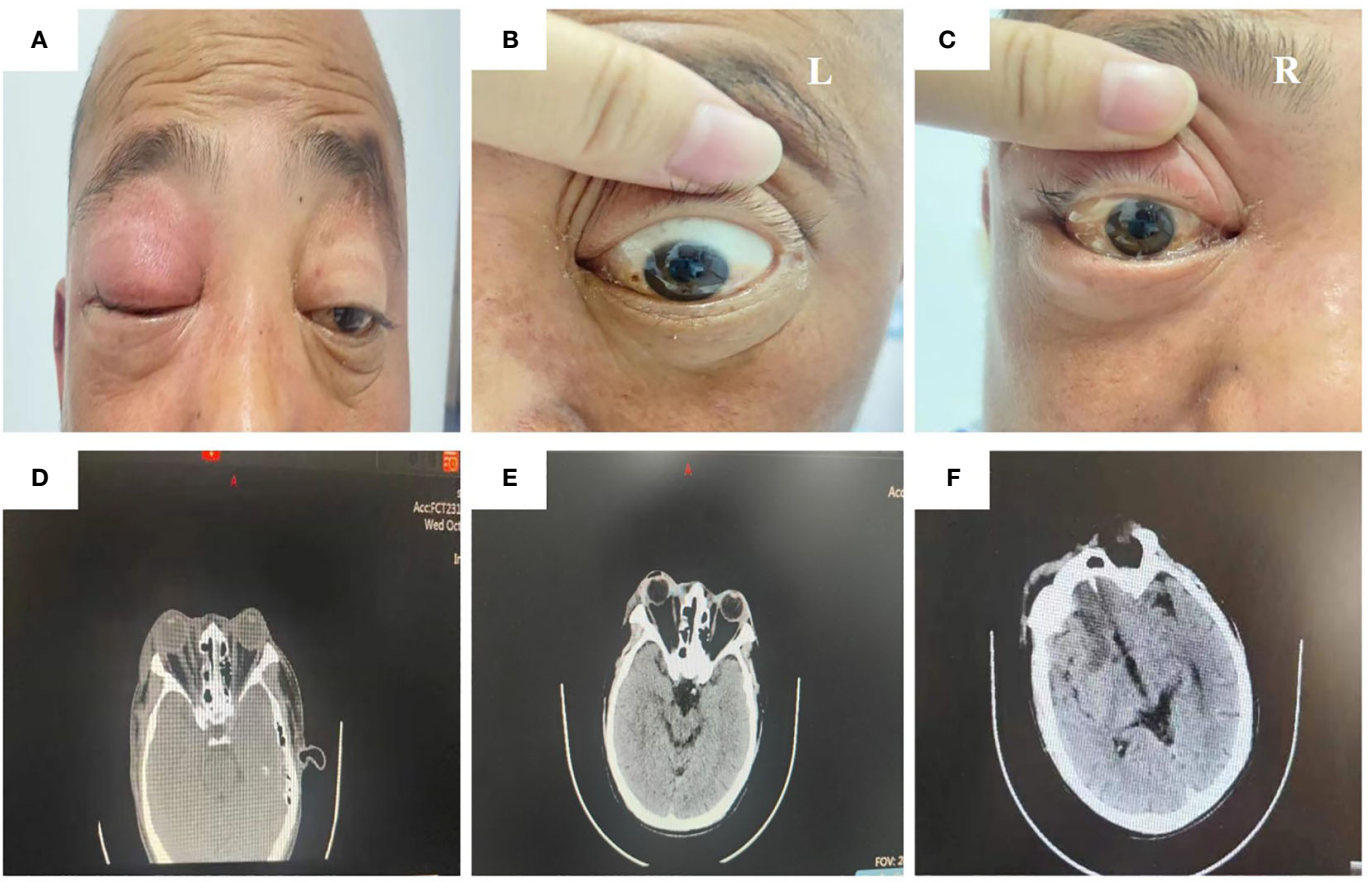
Physical examination and CT image of the patient during administration. The ocular signs of physical examination on day 1 after admission **(A–C)**. The orbital CT image on day 1 after admission showed bilateral maxillary and ethmoid sinusitis **(D)**. The head CT image on day 3 after admission showed swelling of soft tissue around the right orbit, slightly aggravated compared to the first day after admission **(E)**. The head CT image on day 4 after admission showed a low-density shadow in the bilateral frontal lobe and right anterior horn of the lateral ventricle **(F)**.

On admission, his physical examination presented swelling, proptosis, a mid-dilated and fixed pupil, unresponsiveness to light, no light perception, and restriction of extraocular movements of both eyes in all directions (**Figures 1A–C**). Laboratory evaluation revealed a white blood cell count of 18.3 × 10^9^/L with 89% neutrophils. Computed tomography (CT) imaging of the head revealed bilateral maxillary and ethmoid sinusitis (**Figure 1D**). Based on the clinical signs and imaging results, the etiologies were considered inflammatory by multi-disciplinary treatment (MDT), and the patient was administered vancomycin and methylprednisolone sodium succinate after admission.

Nevertheless, the patient’s condition did not improve after the initial treatment. The redness and swelling of the eyelids in both eyes gradually worsened, and the skin from the right orbit and right nasal wing to the tip of the nose was black and necrotic. On the third day after the presentation, the patient began to experience fever (with a maximum body temperature of 39°C) and complained of numbness in the right facial area. The result of a recheck of the head CT revealed the worsening of right eye swelling (**Figure 1E**).

The patient was suddenly subjected to left limb dysfunction and lisps without obvious inducement on the morning of the fourth day after the presentation. The head CT showed low-density shadows in the bilateral frontal lobe and right anterior horn of the lateral ventricle, as well as sinusitis (**Figure 1F**). The laboratory testing reported a white blood cell count of 18.82 × 10^9^/L with 82.9% neutrophils and a PCT value of 1.86 ng/mL. Taking the increasing inflammatory level into consideration, the patient was administered vancomycin and meropenem.

Due to the rapid development of the patient’s condition, mNGS detection using a peripheral blood sample was performed to identify the potential pathogens in a rapid on-site mNGS platform in the hospital. QIAamp UCP Pathogen Mini Kit (QIAGEN, Hilden, Germany) was used for DNA extraction according to the manufacturer’s instructions. The extracted DNA was quantified using a Qubit dsDNA HS Assay Kit (Thermo Fisher, Massachusetts, USA). The library was constructed through DNA fragmentation, end-repair, adapter-ligation, and PCR amplification. Qubit and Agilent 2100 Bioanalyzer (Agilent Technologies, Santa Clara, CA, USA) were used to assess the quality of the DNA library. The qualified double-strand DNA library was transformed into a single-stranded circular DNA library through DNA denaturation and circularization. DNA nanoballs (DNBs) were generated from single-stranded circular DNA using rolling-circle amplification (RCA). The DNBs were qualified using Qubit 4.0. Qualified DNBs were sequenced on the BGISEQ-200 platform using a 50-cycle single-end sequencing strategy.

**Figure 2:**
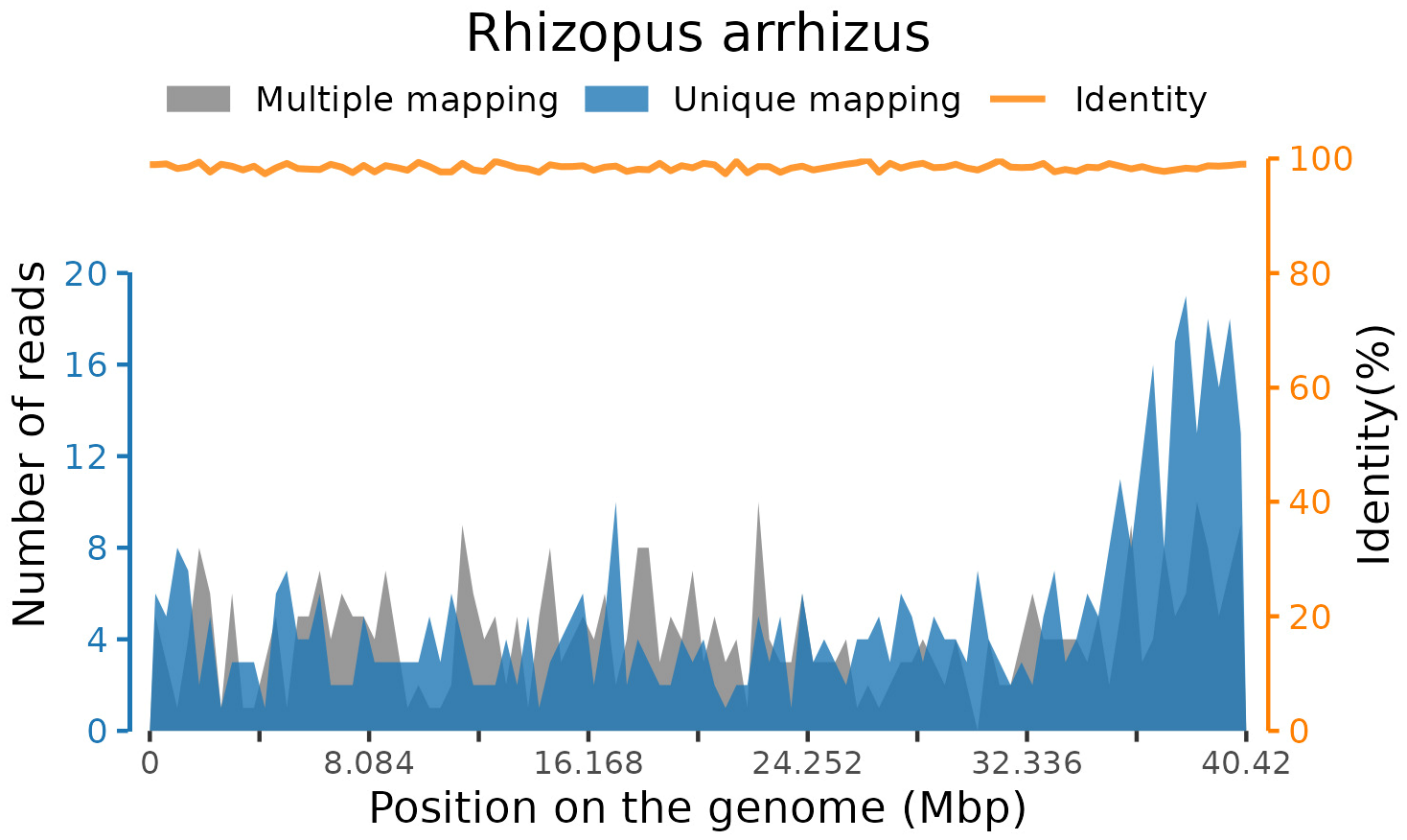
Coverage of *Rhizopus arrhizus* detected by mNGS in a blood sample.

After sequencing, adaptor contamination, low-quality reads, duplicate reads, and low-complexity reads were removed from the raw data using fastp software (Chen et al., 2018) with default parameters. Human DNA was also filtered out by mapping to the human reference genome (hg38) using STAR alignment (Dobin et al., 2013). The remaining reads were then classified by simultaneously aligning with in-house microbial genome databases, consisting of viruses, bacteria, fungi, and parasites associated with human diseases. Finally, 156 standardized species-specific reads from blood samples were uniquely aligned to the *Rhizopus arrhizus* genome (**Figure 2**).

The mNGS result was subsequently confirmed by tNGS testing. Contrary to the mNGS workflow, a predesigned primer pool was added to the extracted nucleic acids to facilitate targeted enrichment of pathogen DNA before the sequencing library construction in tNGS testing.

Combined with the clinical manifestations, the patient was finally diagnosed as ROCM-infected with the fungal *Rhizopus arrhizus*. However, the patient experienced a sudden drop in heart rate and unfortunately died on the night of the fourth day after the presentation.

## Discussion

ROCM, as one of the most devastating manifestations of mucormycosis, is a rare and opportunistic yet highly aggressive and lethal fungal infection that is wreaking havoc at an alarming rate in India and several other countries during the COVID-19 epidemic period (Rudrabhatla et al., 2022; Abdorahimi et al., 2023; B et al., 2023; Erami et al., 2023). The clinical manifestation of ROCM is usually presumed to originate from the nasal mucosa and sinuses, presenting as nasal congestion, runny nose, fever, or headache, then progressively spreading to the orbit, with symptoms such as protrusion of the eyeball, swelling of the eyelids, ptosis of the upper eyelid, restricted eye movement, bulbar conjunctival edema, dilated pupils, and loss of light response, and finally spread to brain tissue within only a few days, ultimately leading to death (Bae et al., 2012; Cornely et al., 2019).

The patient in our report had uncontrolled diabetes, making him in the high-risk group for mucormycosis infection. He first presented in our hospital with vision loss in the right eye. Examination revealed swelling, proptosis, a mid-dilated and fixed pupil, unresponsiveness to light, no light perception, and restriction of extraocular movements of both eyes in all directions,. These findings revealed a RCOM originating from the orbit. Shortly thereafter, a head CT scan showed low-density shadows in both frontal lobes and adjacent to the anterior horn of the right lateral ventricle, suggesting that RCOM had spread to the nervous system, which ultimately led to the patient’s death. It is almost impossible to win over the fungi after they enter the intracranial cavity, with literature evidence of up to 100% mortality in cerebral mucormycosis (Azhar et al., 2022; Rudrabhatla et al., 2022; El Hakkouni et al., 2023).

At present, there are no effective clinical biomarkers for ROCM diagnosis (Walsh et al., 2012; Lass-Florl, 2017). Previous studies have revealed that up to 90% of ROCM cases are undiagnosed and untreated (Walsh et al., 2012; Dong et al., 2022). In our present case, we obtained a rapid and accurate etiological result within 24 h with the help of a novel technology called mNGS, which has emerged as a fast, precise, and effective laboratory technology. Compared with traditional diagnostic methods, the chief advantage of mNGS lies in its unbiased sampling, which enables the simultaneous identification of all potentially infectious agents in samples and avoids defining the targets for diagnosis beforehand. In the current study, we detected *Rhizopus arrhizus* sequences in a peripheral blood sample, and the result was subsequently confirmed by tNGS testing. It was finally diagnosed as ROCM infected with the fungal *Rhizopus arrhizus*, combined with the clinical manifestations. *Rhizopus arrhizus* is a species of mucoromycotan fungi in the family Rhizopodaceae. Findings from previous studies have shown that *Rhizopus arrhizus* is identified as the dominant agent in ROCM patients with diabetes in the Middle East and Asian countries like Iran and India (Guinea et al., 2017; Abdorahimi et al., 2023; Erami et al., 2023). However, few cases have been reported in China currently.

Although the patient had already unfortunately died before the mNGS results were available, taking the rapid progression of ROCM into consideration, it is still highly recommended that mNGS testing be performed as the first choice to identify potential pathogens in suspicious cases.

In conclusion, ROCM is a rare, lethal, and infectious disease that requires early diagnosis and timely treatment for successful therapy. The misdiagnosis rate is very high in the early stage due to the lack of specific clinical features. Prompt diagnosis of ROCM infection with aggressive antifungal therapy is crucial to increasing survival and reducing mortality. It is strongly recommended that rapid mNGS pathogen testing be the first choice for highly suspicious patients, especially for cases with uncontrolled diabetes or immunocompromised ones.

## Data availability statement

The original contributions presented in the study are included in the article/supplementary material. Further inquiries can be directed to the corresponding authors.

## Ethics statement

The studies involving humans were approved by the ethics committee of First Affiliated Hospital of Dalian Medical University. The studies were conducted in accordance with the local legislation and institutional requirements. The participants provided their written informed consent to participate in this study. Written informed consent was obtained from the individual(s) for the publication of any potentially identifiable images or data included in this article. Written informed consent was obtained from the participant/patient(s) for the publication of this case report.

## Author contributions

JL: Writing – original draft. YG: Writing – original draft. CX: Writing – original draft, Methodology. LJ: Writing – original draft, Writing – review & editing.
